# CellProfiler: Novel Automated Image Segmentation Procedure for Super-Resolution Microscopy

**DOI:** 10.1186/s12575-015-0023-9

**Published:** 2015-08-07

**Authors:** Kareem Soliman

**Affiliations:** Department of Pediatrics and Adolescent Medicine, University Medical Center, University of Göttingen, Robert-Koch Str.40 1.D3.644 Histology Lab, Göttingen, 37075 Germany

## Abstract

**Background:**

Super resolution (SR) microscopy enabled cell biologists to visualize subcellular details up to 20 nm in resolution. This breakthrough in spatial resolution made image analysis a challenging procedure. Direct and automated segmentation of SR images remains largely unsolved, especially when it comes to providing meaningful biological interpretations.

**Results:**

Here, we introduce a novel automated imaging analysis routine, based on Gaussian, followed by a segmentation procedure using CellProfiler software (www.cellprofiler.org). We tested this method and succeeded to segment individual nuclear pore complexes stained with gp210 and pan-FG proteins and captured by two-color STED microscopy. Test results confirmed accuracy and robustness of the method even in noisy STED images of gp210.

**Conclusions:**

Our pipeline and novel segmentation procedure may benefit end-users of SR microscopy to analyze their images and extract biologically significant quantitative data about them in user-friendly and fully-automated settings.

## Background

Super resolution (SR) microscopy unlocked new opportunities for cell biologists to investigate cells and cellular functions at unprecedented resolution up to few nanometers, which require re-thinking of biologists about new and previous discoveries [1]. SR microscopy visualizing single molecules clusters at nanometer resolution has made image analysis a more complicated practice. Current image analysis of SR microscopy data rely mostly on complex analytical tools and MatLab (www.mathworks.com) based routines. Automated grouping of molecule clusters into biologically meaningful objects by direct segmentation remains largely difficult; however, density algorithms optimized for Single Molecule Localization Microscopy (SMLM) was the first automated attempt to segment and interpolate object boundaries directly from SMLM images by using local adaptive density kernels to merge and separate molecules clusters into meaningful objects [2].

Image segmentation algorithms that use shape or intensity data will identify single super resolved clusters, but will not perceive a group of separate clusters as one functionally active domain. Automated algorithms that rely on adaptive densities information to segment and interpolate boundaries have been only shown to work well with SMLM on reference structures with continuous densities e.g., mitochondria, microtubule [2], however; computational performance and accuracy on structures with intermittent densities and profiles remains lacking e.g., nuclear pores complex. For such problems, most biological studies involving SR microscopy techniques rely on manually defining regions of interests (ROIs) with geometry that best fits structures investigated e.g., ellipse, circular, square or rectangular [3–5]. This type of manual work is a very tedious job, but most reliable alternative so far.

SR microscopy techniques provide a much narrower Gaussian point spread function (PSF) of the focused scanning area, enabling us to resolve features that are distant below diffraction limit of light, which is approx. about 200–300 nm in (xy) and up to 500–700 nm in (z) axis [6]. Smoothing is applied to images to average signals using a smoothing function. Interestingly, applying a Gaussian could enlarge PSF of SR images and drive resolution backward toward diffraction limit of light in a controlled fashion by mathematical means e.g., increasing width of Gaussian. An intuitive way to segment SR images into meaningful groups of molecules would be through Gaussian smoothing to merge proximate signals within the artifact radius of the Gaussian. Therefore, structures and groups of molecules clusters can be merged when distance separating them is below Gaussian width of the applied filter. Once this cluster grouping is achieved, segmentation remains to be done. Merged super resolved structures might show intensity variation throughout the whole object, because each of the merged clusters has different intensity profile. For that, it is logical to use algorithms that use shape information to segment clumped objects, avoiding by that over-segmentation problems [7].

Once groups of clusters in an image are perceived as biologically significant objects by means of Gaussian blurring, it should remain possible to segment the actual SR image to find out single molecule cluster information in the image. Associating objects found in the SR image and its Gaussian blur, would allow us to find out meaningful data about each group of molecules. Interestingly, automated imaging free ware software tools, like CellProfiler (www.cellprofiler.org), possess all previously described algorithms and analysis paradigms [8], which should make it possible to implement Gaussian blur filters and combine image segmentation procedures to extract meaningful data about clusters of molecules directly from SR images in a fully automated way.

In this paper, we introduce and test a novel image analysis procedure for SR microscopy, which depends on Gaussian blurring to merge super-resolved structural details in SR images into biologically meaningful objects. Followed by a segmentation process, it was then possible to interpolate objects boundaries in blurred images. Relating objects from both SR images and their blurred versions allowed direct reading and quantification of cluster information per groups of molecules. We used simulation data and CellProfiler to explain how the analysis works and we applied our approach to study structures of nuclear pore complex, to show our approach running on real data.

## Results

### Basic Simulation of SMLM Image and Automated Cluster Analysis by Cellprofiler

**Fig. 1:**
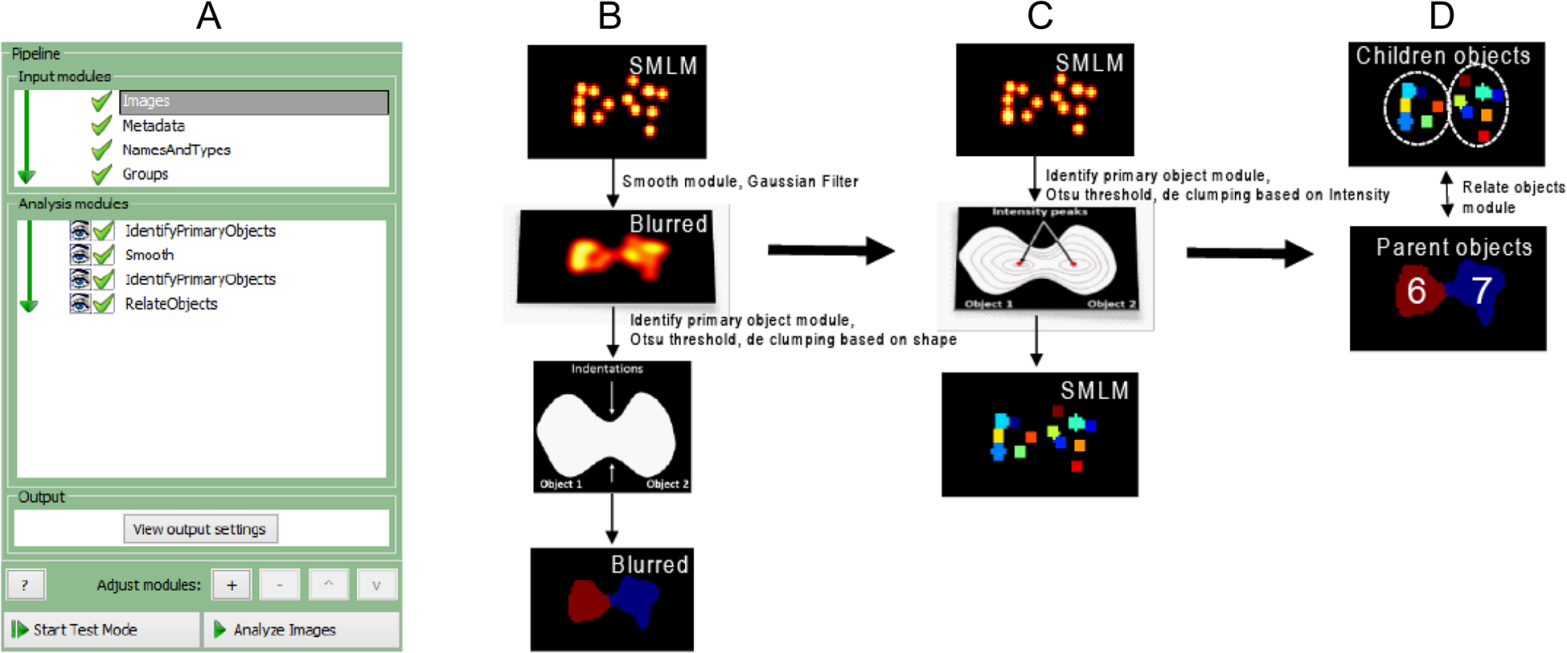
Pipeline and analysis tested on SMLM simulation image. **a** Pipeline design in CellProfiler window. **b** Simulation image blurring, segmentation and de clumping by shape algorithm. **c** Simulation image segmentation and de clumping by intensity watersheds. **d** Relating segmented images before and after blurring with number of virtual bruchpilot clusters per virtual active zone indicated

We created a basic simulation image of two adjacent single molecule clusters that simulate SMLM data e.g., PALM or STORM of the active zone bruchpilot protein clusters at synapse of neuromuscular junctions (NMJs) of Drosophila [3, 9]. We used CellProfiler to automatically count number of clusters per active zone by designing a pipeline tree in (Fig.1b). First, we used a Smooth module to apply a Gaussian filter with an artifact diameter that is capable of merging only nanoscopic clusters of both virtual active zones, while leaving it possible to delineate the two active zones (Fig. 1b). Next, we used “IdentifyPrimaryObject” module and we set MCT thresholding as indicated in materials and methods. De clumping of individual active zones was done by searching for shape indentations in the fused objects (Fig. 1b), while detection of single molecule clusters per active zone was done by another automated “IdentifyPrimaryObject” module to find single molecule clusters (Fig. 1c). In the latter object search module, we used the same thresholding strategy; however, to de clump individual single molecule clusters we used maximum intensity to divide clumped objects (Fig. 1c). Since both identified set of objects or features come from the same image, re-alignment was not required and we could accurately relate objects using “RelateObjects” module. This made it possible to quantitatively count number of clusters per active zone (Fig. 1d).

### Automated Counting of gp210 Subunits and pan-FG Protein per Nuclear Pore Sites Imaged By STED Microscopy

**Fig. 2:**
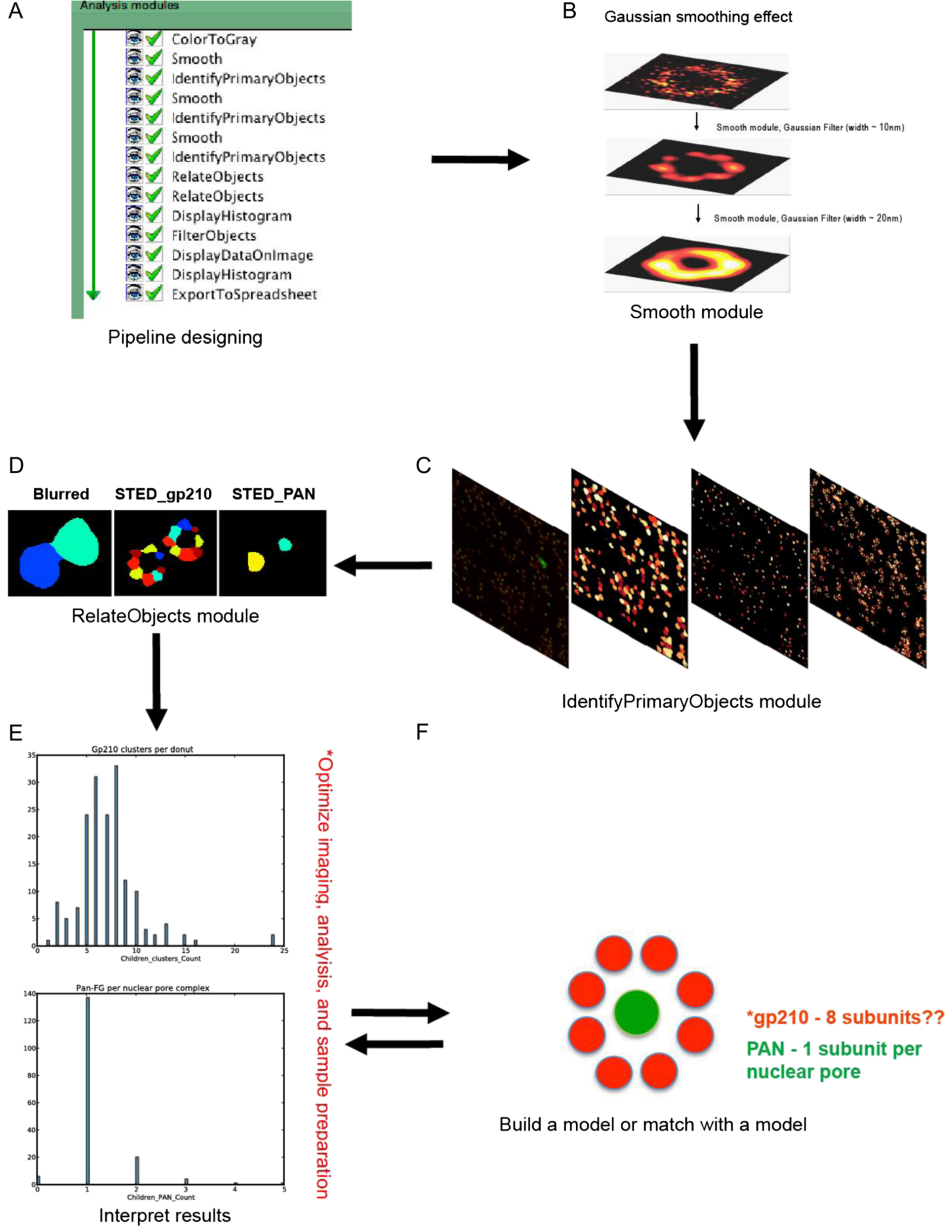
Automated counting of gp210 and pan-FG protein clusters per nuclear pore complex. **a** Overview showing pipeline modules sequence. **b** Influence of Gaussian blurring on structure of gp210 STED resolved structures. **c** Two color raw STED image of immunostained gp210 (red) and pan-FG (green) and images series produced after segmentation. **d** Zoom in image of segmentation results from CellProfiler window showing two neighbouring nuclear pores (left), gp210 clusters (middle), and pan-FG clusters (right). **e** Histogram plots showing quantitative segmentation results. **f** Current model of nuclear pore complex and two direction arrows connecting models with experimental results

We tested the performance of our analytical approach on STED images of gp210 and pan-FG proteins. Gp210 protein is known to be the main cause for the symmetrical eight fold structures around the central channel of nuclear pores, whereas pan-FG labels the central channel with an average of one cluster per complex [10, 11]. We designed a pipeline to extract number of gp210 and pan-FG clusters per nuclear pore complex directly from STED images using our novel approach (Fig. 2a). Using imageJ “Gaussian Blur” plug-in, we confirmed that Gaussian smoothing de blur noisy signals in fluorescence images (Fig. 2b). Interestingly, expanding Gaussian diameter by a factor more than the resolution of super resolved gp210 clusters, we were able to merge adjacent gp210 subunits of nuclear pore complex into donut structure (Fig. 2b). In the automated pipeline, we applied a sequence of Gaussian filters to detect single nuclear pore structures (donuts) and their respective gp210 clusters and used MCT threshold algorithm for both donuts and gp210 clusters. However, we used maximum intensity watersheds and shape to de clump gp210 clusters and their donuts, respectively (Fig. 2c). To identify pan-FG protein, we applied a Gaussian of 5.0 pixels and shape was used to de clump objects (Fig. 2c). Relating all objects (gp210 clusters, gp210 donuts, and pan-FG clusters), we resolved that each nuclear pore complex consist of an average of 1 pan-FG protein, and histogram data showed highest frequency at eight gp210 clusters per nuclear pore (Fig. 2d and e). We also suggest a more global experimental and analytical scheme (Fig. 2e and f) that takes into account the following:

1. Modeling geometry of structures of interest, their clustering density and relative orientation of clumped objects all of which decides parameters and stringency of Gaussian and segmentation algorithms.
2. Optimization of imaging acquisition parameters e.g., finding best field of view, right detector pixel size and laser power.
3. Control experiments to optimize biophysical related parameters e.g., labeling density optimization, label size, and label specificity.
4. Careful testing of models for any type of quantitative SR microscopy experiment and increasing (n) number of images to calculate statistical significance.

**Fig. 3:**
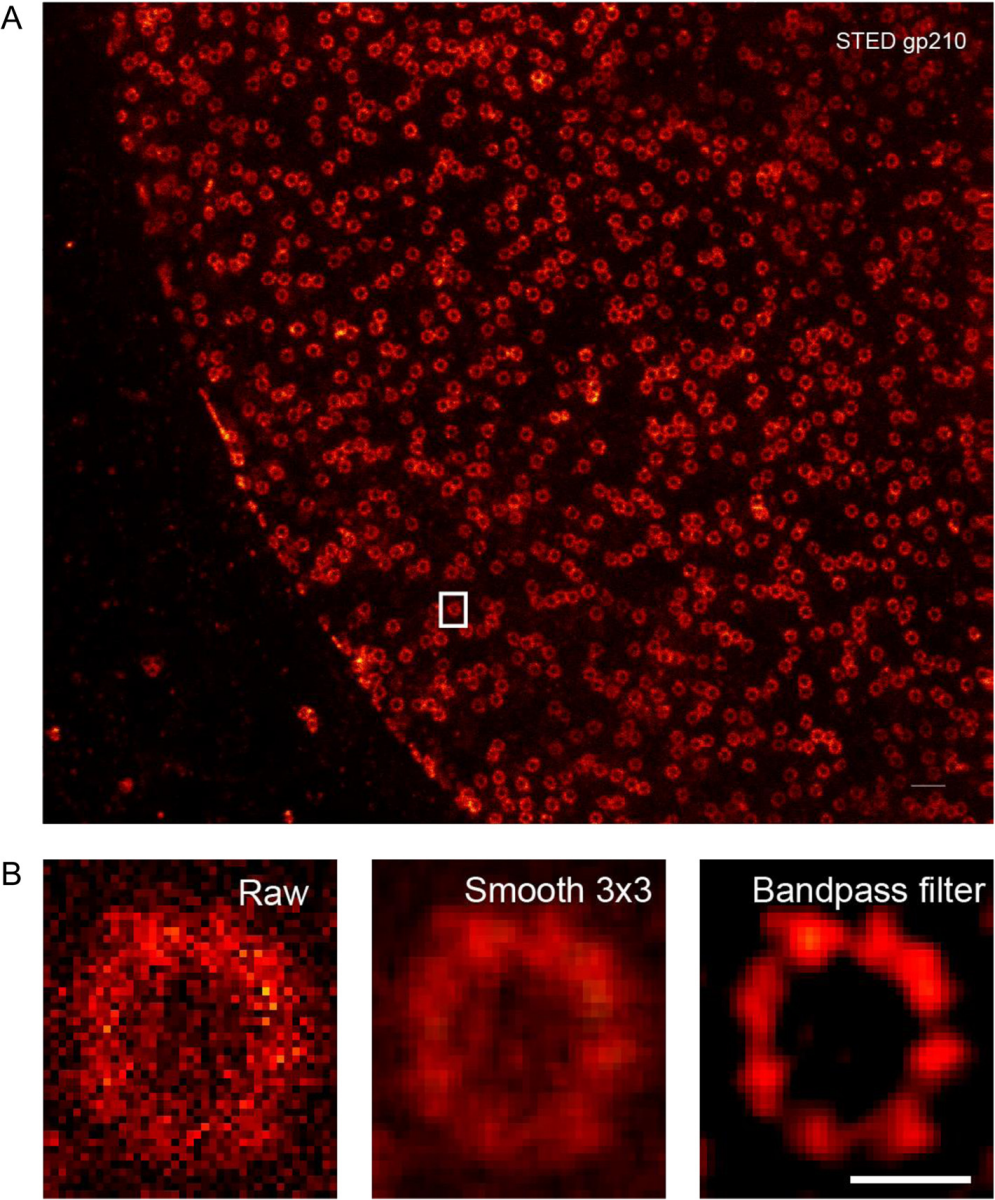
Enhancing image contrast by band pass filter improves visualization of gp210 clusters in noisy STED images. **a** Overview of single color STED image of gp210. **b** Close up on one nuclear pore (left), signal was enhanced by 3x3 smoothing (middle), or contrast enhanced by bandpass filter (right). Scale bar 500 nm in (**a**) and 100 nm in (**b**)

**Fig. 4:**
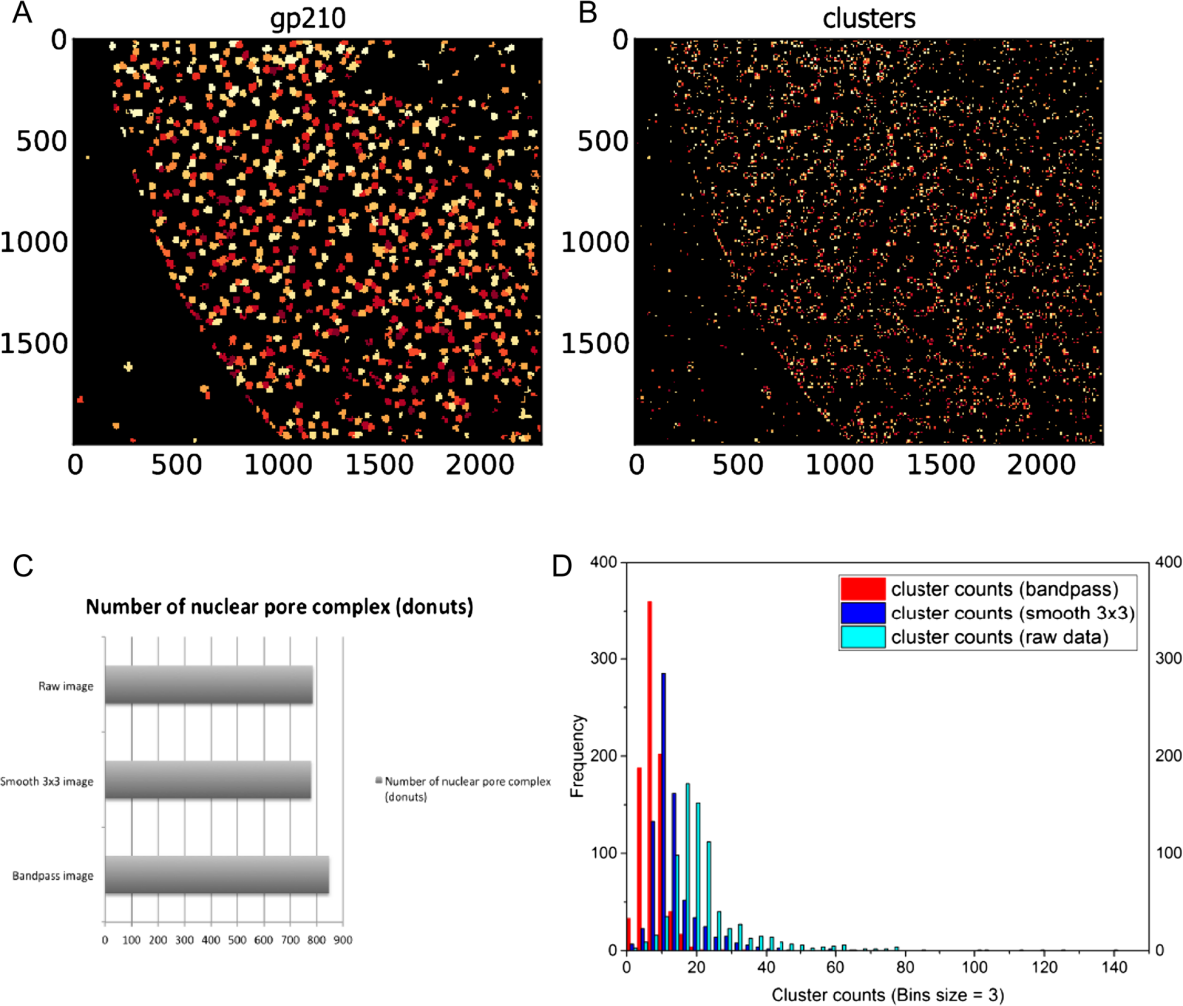
Comparing segmentation results on raw STED image, with and without 3x3 smooth or band pass filters. **a, b** Segmentation image of gp210 donuts (**a**) and gp210 clusters (**b**). **c** Number of donuts segmented from raw image, smoothed image, and band pass filtered image. **d** Over segmentation artifacts of gp210 clusters per gp210 donuts significantly reduced in image pre-processed by a bandpass filter using ImageJ

Further, we used a noisy single color STED image of gp210 (Fig. 3a). To enhance visualization of individual gp210 subunits, we applied a smooth filter in ImageJ to enhance features signals, but it was not sufficient and for that we used a bandpass filter to reduce edge pixels artifacts and enhance contrast (Fig. 3b). (Fig. 4a and b) show an example of segmented images. Intriguing quantification results revealed that bandpass filter, not smoothing, was sufficient to boost the accuracy of cluster segmentation and we had a peak value of 8 clusters per donut from that image (*n* = 167 show 8 subunits per nuclear pore from an *n*_*total*_ = 847), while over segmentation was prominent in raw image and even after smoothing; as indicated by shifting of the peak of the histograms toward the right i.e. more clusters per donuts (Fig. 4d). No evident change in the segmentation of donuts before and after preprocessing (Fig. 4c). Our final results are in agreement with previous reports of super resolution images of gp210 and pan-FG protein.

## Discussion

Manually defining and tracking biologically meaningful ROIs in images obtained by SR microscopy is time consuming and predominantly subjective task; however, it is still the most commonly used approach in SR image analysis [3–5, 12]. We designed pipelines to perform automated quantitative analysis on images acquired by SR microscopy based on a novel procedure, which involves Gaussian fusion of super-resolved clusters into meaningful objects. We succeeded to directly perform cluster analysis on nuclear pore complex proteins by overlying Gaussian fused objects (donuts) with segmentation results from the original SR images (gp210 clusters) to estimate number of gp210 clusters per nuclear pore.

Our method maybe potentially promising for many cell biologists who want to extract quantitative data from their SR microscopy data, imposing a great advantage that it can be run on fully automated mode via CellProfiler and does not require special computer programming skills. Even though we have not presented test results on other interesting cellular structures, we propose that our method and the scope of application will only grow by testing it on more biological structures tackling different interesting research questions that remain to be answered by high resolution quantification analysis; partly we are doing this in our research with collaboration and we are obtaining promising results for biological discoveries.

In this paper, we mainly report the novelty and creativity of the procedure and to make pipelines available for public to use, modify and develop. We also suggest that image pre-processing e.g., applying bandpass filters, and critical inspection of SR images quality should always be carefully considered, clearly stated how it was carried out before publishing any quantification results. We predict that future advancements in the field of user-friendly automated image analysis solutions optimized for SR microscopy will follow.

## Conclusion

The proposed computational image segmentation procedure is a novel method optimized for super resolution microscopy. It could allow direct segmentation of super resolved features in fully automated and user-friendly settings. We successfully quantified the composition of the amphibian nuclear pore complex stained by gp210 and pan-FG proteins imaged with STED microscopy and CellProfiler was used to implement our method in fully automated mode.

## Methods and Materials

### ImageJ and SMLM Data Simulation

Image Gaussian filters applied in (Fig. 2) was done using ImageJ “Gaussian Blur” Plug-in. SMLM simulation in (Fig. 1) was made by brush tool in ImageJ to draw single molecule clusters that resemble Bruchpilot protein clusters at Drosophila neuromuscular junction (NMJ) active zones [3].

### STED Images and Pre Processing

STED images used in (Figs. 2, 3 and 4) of gp210 and pan-FG proteins of nuclear pore complex in ovarian amphibian cells were imaged by a two color STED microscope and were valuable gifts from Fabian Göttfert (Prof. Stefan Hell’s Lab) [11]. Pre-processing of STED images via smoothing 3x3 pixels averaging, bandpass or Gaussian filters, and linear intensity scaling (for clear visualization) were done in ImageJ and all images were stored as tiff colored (RGB) formats.

### CellProfiler Pipelines and Data Analysis

We used CellProfiler (version 2.1.1) to design and execute pipelines used in this paper. Gaussian filter was used for smoothing images by running smooth modules. Filter artifact diameter was set according to requisite effect, either to merge groups of structures/molecules into biologically meaningful ensembles, or to blur out noise to aid segmentation. “IdentifyPrimaryObject” module was used to find objects of interest in the analyzed images. Unless stated otherwise, Maximum Correlation Thresholding (MCT) was implemented with 1 pixel Gaussian smoothing [13]. Separating clumped objects was carried based on shape of objects or maximum intensity values within a radius range depending on objects of interest. Distance for lower maxima suppression, which aid to separate clumped objects was set manually to median radius of objects sizes. In that way most of clumped objects in test images did not display over segmentation artifacts. To associate segmented objects found in SR images, their respective blurred copy, or any images in different channels in case of two color imaging, we applied “RelateObjects” automated module. According to analysis purpose we set objects as either ‘parents’, or ‘children’ and data were plotted directly by “DisplayHistogram” modules. Data were also exported to spreadsheets using “ExportToSpreadsheet” module to use them in further analysis and to plot data. We used OriginPro9.1 to plot data.

## Abbreviations

SR: Super resolution
STED: Stimulated Transmission Emission Depletion
ROI: Region of interest
Gp210: Glycoprotein 210
SMLM: Single Molecule Localization Microscopy
PALM: Photo Activatable Light Microscopy
STORM: Stochastic Optical Reconstruction Microscopy
